# Sex as a biological variable in amblyopia: implications for developmental plasticity and treatment

**DOI:** 10.3389/fnins.2026.1846268

**Published:** 2026-05-04

**Authors:** Kathryn M. Murphy, Leanne Monteiro, Maheshwar Panday, Nadine Barone

**Affiliations:** 1 Department of Psychology, Neuroscience and Behaviour, McMaster University, Hamilton, ON, Canada; 2 McMaster Neuroscience Graduate Program, McMaster University, Hamilton, ON, Canada

**Keywords:** amblyopia, critical period, developmental plasticity, patching therapy, sex differences, sex-specific, treatment response, visual cortical plasticity

## Abstract

Amblyopia is a common childhood visual disorder caused by abnormal visual experience that drives visual cortical plasticity during the sensitive period. The timing and forms of treatment with patching therapy and other therapeutic interventions have been extensively studied; however, sex has not been a primary focus in studies examining amblyopia. This mini-review synthesizes evidence for sex differences across levels of analysis, from typical visual development to animal models of amblyopia and human studies of amblyopia incidence and treatment. In addition, it highlights latent sex differences in plasticity mechanisms that may provide insights for future visual neuroscience studies of amblyopia. These findings highlight a key concept: visual outcomes may appear similar yet depend on different mechanisms in females and males, potentially influencing the durability of recovery. We discuss a framework to advance a sex-aware research pipeline for amblyopia, spanning basic, preclinical, and clinical/translational research.

## Introduction

Abnormal visual experience early in life, such as strabismus or unequal refractive error, can lead to persistent deficits in visual acuity, contrast sensitivity, and binocular vision, the hallmarks of amblyopia. Amblyopia is distinctive among neurodevelopmental disorders in that multiple effective treatments are available, and a large experimental literature has identified key neurobiological mechanisms that regulate its development and experience-dependent plasticity. Amblyopic visual deficits are cortical in origin and have historically been studied within a framework that prioritizes experience-dependent plasticity and the age of treatment, while minimizing, and in some cases overlooking, other sources of variability that might influence treatment response and clinical care of the whole child (Birch and Kelly, 2023). As a result, sex and other host factors (e.g., socioeconomic status, ethnicity) have typically been treated as variables to be controlled rather than as factors to be studied. Notably, among the top 100 most-cited papers in amblyopia research (Allon et al., 2023), few human studies and none of the animal studies examined sex differences.

This approach has been highly successful in defining sensitive periods, characterizing deprivation-induced reorganization of the visual cortex, and establishing optimal timing for effective treatment during early development. However, it has also produced a literature in which the absence of reported sex differences may be interpreted as evidence that sex does not influence amblyopia vulnerability or recovery. Importantly, this conclusion rests largely on study designs that explicitly balance or match participants by sex, thereby reducing sex-related variance and rendering sex effects difficult to detect unless they are extremely large.

In parallel, research in other neural plasticity domains has demonstrated that distinct molecular, cellular, and circuit mechanisms can support equivalent behavioral outcomes in females and males (Box 1). In systems ranging from hippocampal learning and memory to pain processing, sex differences often emerge not at the level of overt behavior, but in the timing, regulation, and biological substrates of plasticity. These findings challenge the assumption that behavioral similarity implies mechanistic equivalence and raise the possibility that sex-dependent developmental programs may also shape plasticity in the visual cortex.

Box 1 **Latent sex differences in hippocampal synaptic plasticity and memory.**

**Figure 1:**
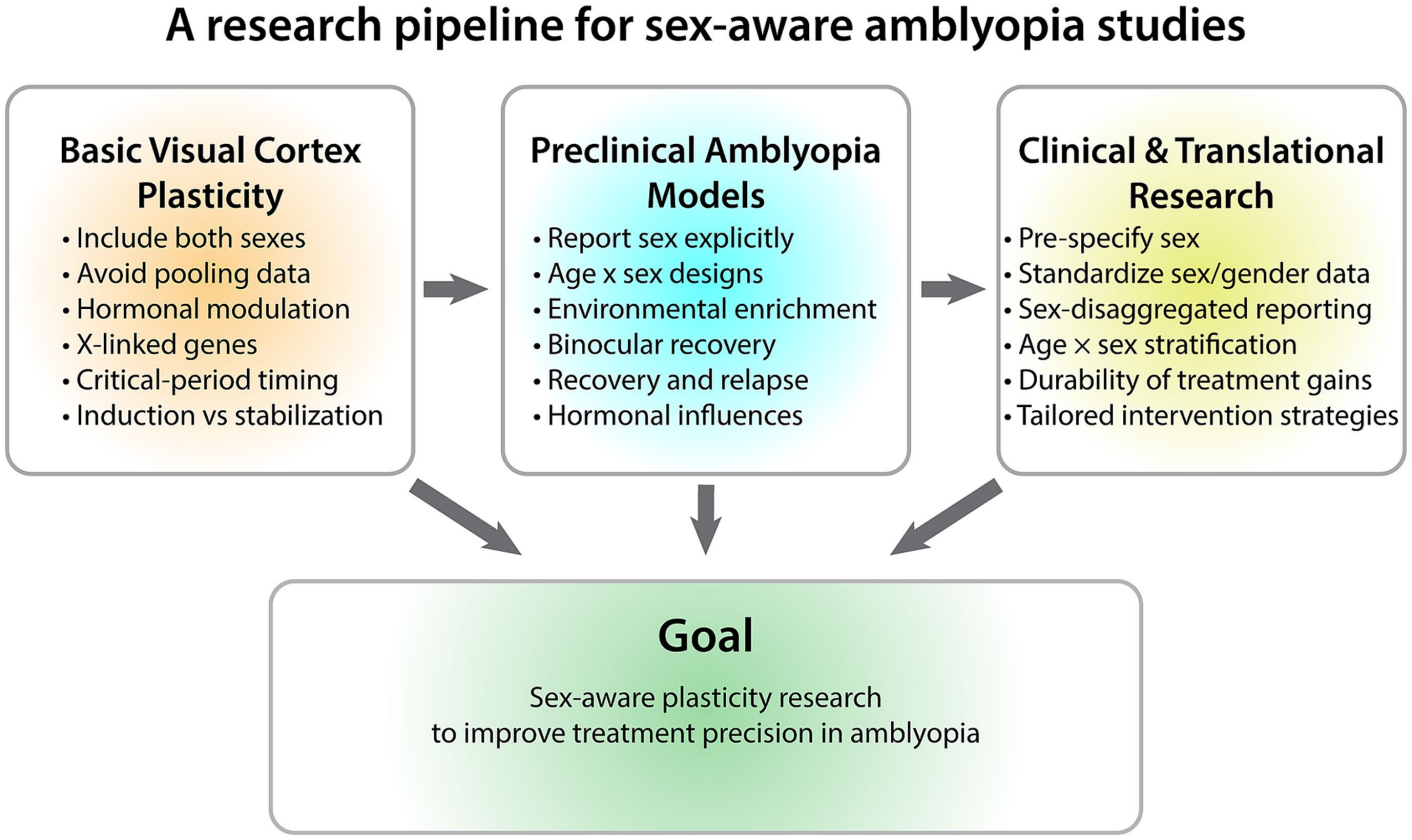
Sex-aware research pipeline for amblyopia. Integrating sex-aware approaches across the research pipeline—from basic studies of visual cortical plasticity to preclinical models and clinical investigations—can help clarify how biological variability influences visual plasticity and treatment outcomes in amblyopia.

Studies of visual cortex and hippocampal plasticity have historically converged on a shared set of molecular mechanisms regulating experience-dependent synaptic modification and learning (Bear, 2003). As in the visual cortex, hippocampal plasticity has often been assumed to operate similarly in females and males when behavioral outcomes are equivalent. However, the two studies highlighted below provide compelling evidence of *latent sex differences* in which females and males achieve similar functional endpoints, such as long-term potentiation or memory performance, through distinct underlying molecular and cellular mechanisms. Box Figure 1 illustrates this principle conceptually and summarizes the molecular pathways identified in the two studies.

These findings are relevant to amblyopia research because they demonstrate that behavioral equivalence does not guarantee mechanistic equivalence (Box Figure 1). In the hippocampus, sex differences became evident only when investigators moved beyond outcome measures to examine the specific signaling pathways that support plasticity. This work provides precedent for the possibility that comparable visual outcomes in girls and boys could mask sex-specific mechanisms of plasticity in visual cortex, particularly those related to the timing, regulation, or stabilization of experience-dependent changes.

Study 1: Sex-specific AMPAR mechanisms underlying estradiol-induced potentiation

The study by Jain and Woolley (2023) examined how 17β-estradiol (E2) modulates excitatory synaptic strength in the hippocampus. Although E2 is synthesized locally in both females and males and induces an equivalent magnitude of synaptic potentiation in both sexes, the mechanisms by which that potentiation is expressed and stabilized differ fundamentally.

In females, expression of E2-induced potentiation required ongoing synaptic activity, and stabilization depended on the transient incorporation of calcium-permeable AMPA receptors (cpAMPARs). At the synapse level, most female synapses (~76%) increased strength by increasing AMPAR conductance.

In contrast, in males, potentiation expression did not require synaptic activity, and cpAMPARs were largely unnecessary for stabilization. Most male synapses (~60%) potentiated through changes in non-conductance AMPAR properties, such as channel number or open probability.

Study 2: Sex-specific signalling pathways supporting LTP consolidation and episodic memory)

The study by Le et al., (2024) identified a divergence in how females and males stabilize hippocampal long-term potentiation (LTP) and encode episodic memory, focusing on the role of ion flux-independent (metabotropic) signaling.

In both sexes, NMDAR-mediated calcium influx was required for LTP induction. However, the mechanisms diverged during consolidation. Stabilization of potentiation through actin polymerization was mediated by distinct molecular triggers: males depended on metabotropic signaling via the GluN2B subunit of the NMDAR, whereas females bypassed GluN2B signaling and instead relied on estrogen receptor-α (ERα).

These molecular differences had clear behavioral consequences. Blocking GluN2B signaling disrupted episodic “Where” memory acquisition in males but not females, whereas blocking ERα signaling disrupted the same task in females but not males. Under baseline conditions, females and males performed equally well, despite relying on distinct molecular anchors for memory formation.

Why these studies matter for amblyopia research

Together, these hippocampal studies provide a clear precedent for latent sex differences, in which equivalent plasticity and behavioral outcomes are supported by distinct molecular and cellular pathways (Box Figure 1). In amblyopia research, treatment success is typically evaluated using behavioral or physiological endpoints such as visual acuity or visual evoked potentials, and these hippocampus studies underscore the possibility that similar outcomes in females and males may not reflect identical underlying mechanisms. Rather than implying direct mechanistic equivalence across systems, these studies motivate a more cautious interpretation of null sex effects in amblyopia and highlight the value of probing underlying plasticity pathways when considering the timing, regulation, and durability of treatment-induced recovery.

**Box Figure 1:**
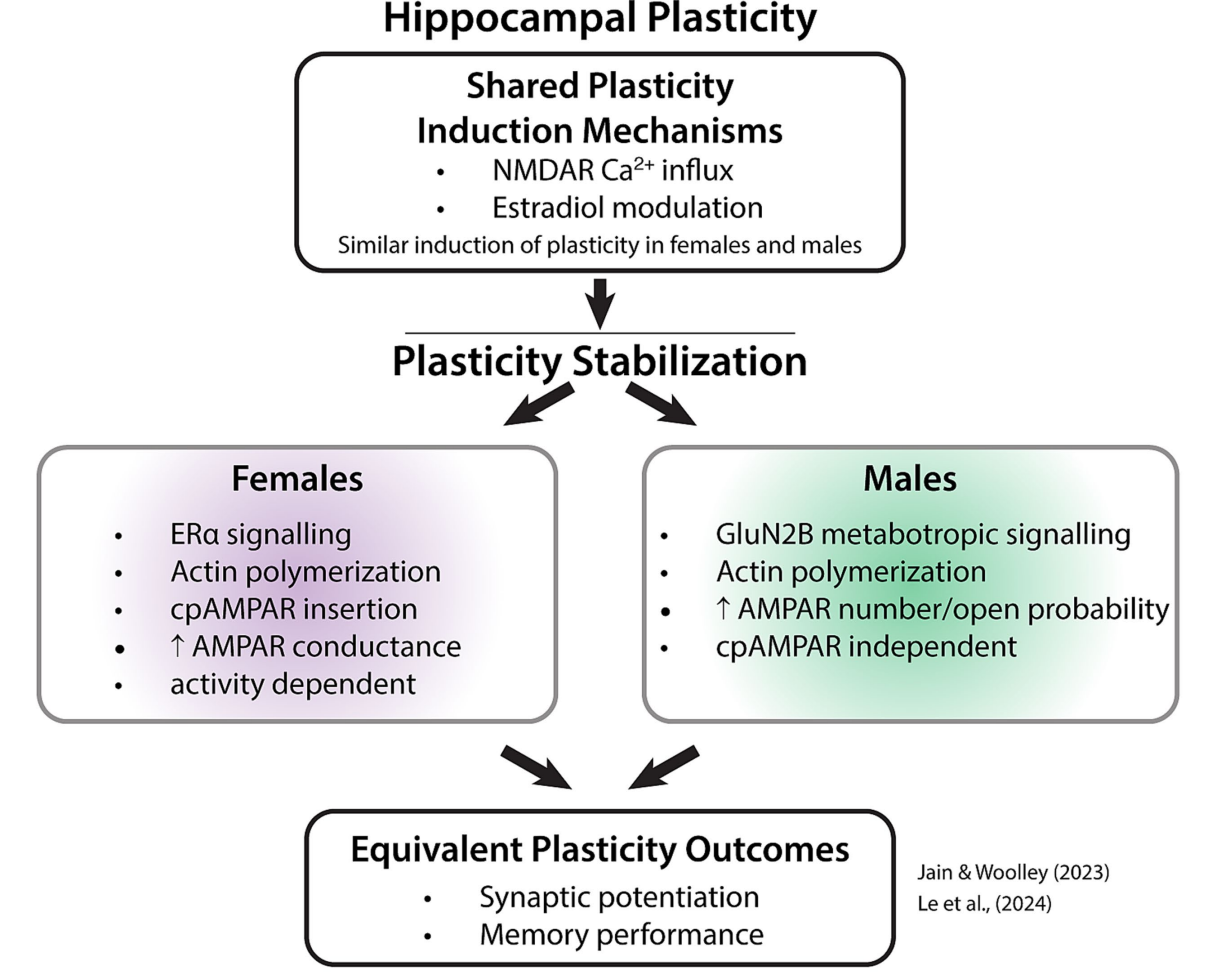
Two studies of hippocampal synaptic plasticity demonstrate that females and males can achieve similar plasticity and behavioural outcomes through distinct molecular pathways. These findings illustrate how equivalent functional outcomes may mask sex-specific mechanisms, highlighting the importance of examining plasticity pathways when considering visual cortical plasticity and recovery in amblyopia.

This Mini Review argues that amblyopia represents a critical gap in sex-aware developmental neuroscience. We contrast the historical treatment of sex in amblyopia research with approaches taken in other neural systems, summarize what is known about sex differences in typical visual development during the sensitive period, and highlight how classic amblyopia study designs constrain inference about sex-dependent mechanisms. We then draw lessons from other neural systems to propose testable hypotheses regarding how sex may influence the timing, mechanisms, and durability of amblyopia treatment effects.

By reframing sex not as a confound to be controlled, but as a potential modifier of developmental plasticity, this review aims to provide a conceptual foundation for next-generation amblyopia studies aligned with contemporary standards in developmental and translational neuroscience. To develop this perspective, we first examine evidence for sex differences in typical visual development, then consider findings from animal models and human amblyopia studies, and finally outline priorities for integrating sex-aware approaches across the research pipeline from basic visual neuroscience to clinical studies.

## Sex differences in normal visual development

There is evidence for sex differences in visual function and visual development across the lifespan. Visual perception and cognition are emergent processes that depend on distributed cortical networks beginning in the primary visual cortex. Because vision is the dominant sensory modality in humans, even modest sex-dependent biases during early development could plausibly influence long-term perceptual and cognitive trajectories. Although most studies of typical visual development do not treat sex as a primary variable, several have reported measurable differences across infancy and childhood.

### Infancy

Studies of infant development suggest early divergence in specific aspects of low-level vision. Binocular vision has been reported to emerge earlier in females than in males (Held et al., 1980), and vernier acuity has been reported to develop earlier in females (Gwiazda et al., 1989). In contrast, males have been reported to exhibit higher contrast sensitivity in early infancy (Dobkins et al., 2009). Electrophysiological measures also suggest potential differences in the tempo of maturation. Pattern-reversal visual evoked potentials (VEPs) show earlier peak latency in infant females (Malcolm et al., 2002), a finding interpreted as reflecting differences in maturation rate, conduction properties, or cortical responsiveness.

These early sex differences in visual sensitivity and physiology are modest. Still, they suggest that the activity patterns driving experience-dependent refinement of visual circuits may differ between females and males even before any signs of clinical amblyopia are detectable.

### Preschool and school-age development

In preschool and school-aged children, sex differences have been reported in several large studies of stereoacuity. Girls showed small but consistent advantages in stereoacuity measured using Randot or Titmus tests (Read et al., 2019; Potluri et al., 2021; Xiao et al., 2025), and male sex has been identified as a risk factor for subnormal stereoacuity in at least one large cohort (Xiao et al., 2025). However, other studies with smaller samples did not detect significant sex differences in visual acuity or stereoacuity (Schmid and Largo, 1986; Han et al., 2019).

Taken together, these findings indicate that sex differences in typical visual development are subtle, task-dependent, and generally small in magnitude. However, even small effect sizes during sensitive developmental windows may be biologically meaningful, particularly if they reflect differences in maturation tempo or in the regulation of plasticity that could interact with experience-dependent change.

## Animal studies

Animal studies of visual cortex (V1) development and plasticity have been central to defining the neurobiological mechanisms underlying amblyopia and its treatment (Hensch and Quinlan, 2018; Birch and Duffy, 2024). However, sex has historically received limited attention in this literature, and the classic studies of V1 development and plasticity were not framed around sex. Many studies implicitly assume that early visual experience shapes V1 development similarly in females and males. Until relatively recently, most animal studies did not report sex, and in mouse models it was common practice to use only males. Although more recent studies include both sexes, data are often pooled.

This gap is notable given evidence of sex-specific plasticity in other neural systems (Box 1) and biological factors that could introduce sex-dependent effects in the visual system. Across species, the male visual cortex is modestly larger than the female cortex, even after adjusting for overall body size (Reid and Juraska, 1992b; Lotze et al., 2019). At the cellular level, V1 expresses receptors for sex and stress hormones, including estrogen and testosterone (Nuñez et al., 2003), as well as enzymes that can locally regulate hormone levels (MacLusky et al., 1986; Clark et al., 1988). In addition, some genes implicated in experience-dependent plasticity are located on the X chromosome and may escape X-chromosome inactivation in females. Despite these considerations, relatively few animal studies have directly examined sex differences in the development or plasticity of V1.

### Evidence of sex differences in V1

Although most animal studies did not explicitly address sex, a series of investigations led by Janice Juraska provided evidence of anatomical and functional differences between female and male rats. These studies reported that:

- V1 is larger in males (Reid and Juraska, 1992b).
- The binocular region contains more neurons and synapses in males, largely reflecting its greater overall size (Reid and Juraska, 1992a, 1995; Nuñez et al., 2001).
- Male rats exhibit better vernier acuity than females (Seymoure and Juraska, 1997).
- Rearing environment can differentially affect dendritic morphology in females and males (Juraska, 1984).

These findings suggest that sex differences in V1 structure and visual performance exist even under typical developmental conditions and may interact with environmental experience.

### Sex and V1 plasticity

Some studies have examined sex as a variable in visual cortical plasticity. For example, housing adult mice in enriched social environments has been shown to produce greater ocular dominance plasticity in females than males (Balog et al., 2014). Sex and estrous cycle stage have also been reported to influence stimulus–response plasticity (Schecter et al., 2023) and the expression of plasticity-related receptors on inhibitory interneurons in V1 (Picard et al., 2019). Furthermore, dendritic spine density is lower in V1 of female than in male juvenile mice (Parker et al., 2020).

Relatively fewer studies have examined sex differences in experience-dependent plasticity during the classical critical period. However, early-life stress paradigms provide suggestive evidence. Repeated maternal separation or early-life chronic stress has been reported to result in retention of juvenile-like ocular dominance plasticity into adulthood in females but not in males (Liu et al., 2020; Li et al., 2022). These findings indicate that sex may modulate how early environmental perturbations shape the temporal boundaries of plasticity.

## Studying sex in human amblyopia research

### Epidemiology

The most common risk factors for amblyopia are strabismus and significant refractive error, including anisometropia (Wallace et al., 2018). Many population-based studies examining the prevalence of these risk factors in children have not identified consistent sex differences (e.g., Wang et al., 2021; Xu et al., 2022). However, a recent large-scale study of more than 9,000 children reported that female sex was associated with an increased risk of anisometropia (Zhou et al., 2023), suggesting that sex-dependent patterns of amblyogenic factors may emerge in sufficiently powered cohorts.

Sex is not widely recognized as a primary risk factor for amblyopia itself. Nonetheless, a meta-analysis of 20 studies reported a modestly higher prevalence of amblyopia in boys than girls (Hu et al., 2022). Although the magnitude of this difference was small and heterogeneity across studies was substantial, these findings indicate that sex differences in amblyopia prevalence should not be entirely dismissed.

Taken together, the epidemiological literature suggests that sex is not considered a dominant risk factor for amblyopia, yet large datasets reveal subtle and sometimes inconsistent sex-associated patterns in both refractive risk and amblyopia prevalence. These mixed findings underscore the importance of distinguishing between the absence of strong effects and the absence of investigation. To better understand how sex might influence amblyopia outcomes, it is useful to consider three related questions: whether sex affects treatment response, whether it influences the durability of treatment gains, and how study design shapes our ability to detect such effects.

### Treatment response

Whether sex influences not only amblyopia risk but also responsiveness to treatment remains an open and underexplored question. Most studies of treatments for amblyopia include both girls and boys, and some have examined the impact of sex as an interaction with the treatment protocol but have not found consistent effects on treatment responsiveness (e.g., Holmes et al., 2003; Repka et al., 2004; Wallace et al., 2006).

For example, a multicenter randomized trial comparing 2 h of daily patching plus spectacles with spectacles alone in children aged 3–7 years with moderate amblyopia (associated with strabismus, anisometropia, or both) found no statistically significant sex × treatment interaction (*p* = 0.07) (Wallace et al., 2006). Although interaction terms were tested within an ANCOVA framework, the primary objective of the trial was to estimate the main effect of patching on short-term (5-week) visual acuity.

In this study design, sex is typically evaluated only through interaction terms or as a predefined covariate in models optimized to detect treatment efficacy. Such models implicitly assume that any sex-related modification of treatment effect is uniform across ages, amblyopia etiologies, and clinical sites. Moreover, the design philosophy of many amblyopia trials has historically prioritized minimizing variability due to host factors, including age, sex, and baseline severity, to isolate treatment effects (Holmes, 2015). Within this framework, sex is treated primarily as a potential confound rather than as a biological variable of interest. Consequently, the studies focus on assessing treatment efficacy rather than testing sex as a moderator of treatment response. Rather than demonstrating that sex does not influence treatment response, such findings are more appropriately viewed as inconclusive with respect to sex as a moderator.

### Stability of visual recovery

Across neural systems, a recurring pattern emerges: plasticity induction can be similar across sexes, but the mechanisms of stabilization or consolidation often differ (Box 1). This distinction is particularly relevant for amblyopia because treatment involves leveraging plasticity in the visual cortex through altered visual experience and examining the durability of the changes. If sex-dependent mechanisms influence how those changes are stabilized, differences may emerge not during the initial treatment response but during maintenance of recovery or susceptibility to regression.

For this reason, studies examining the durability of treatment gains and the risk of regression after therapy may provide a more sensitive window for detecting sex-dependent effects in amblyopia. Earlier studies found that regression after treatment stops is a common outcome (Holmes et al., 2004), but none identified sex as a factor (Holmes et al., 2007). In contrast, a recent retrospective cohort study of children successfully treated for anisometropic amblyopia identified sex as one of the three strongest predictors of visual acuity regression within 1 year, with boys at higher risk of regression than girls (Jia et al., 2022).

The differing conclusions regarding sex effects in visual recovery and recurrence may reflect differences in study design and analytic goals rather than contradictory biological findings. The retrospective cohort study by Jia et al. examined predictors of visual acuity regression after successful treatment and therefore modeled sex as one of several candidate variables influencing the durability of treatment gains. In contrast, randomized treatment trials such as those conducted by the Pediatric Eye Disease Investigator Group (e.g., Holmes et al., 2003, 2007; Repka et al., 2004; Wallace et al., 2006) were designed primarily to estimate the main effect of a treatment protocol on visual acuity outcomes and durability. As a result, studies optimized to detect treatment efficacy may have limited sensitivity for identifying sex-dependent differences in treatment durability or recovery trajectories. Together, these differences highlight how the framing of research questions and analytic strategies can shape whether potential sex effects are detectable in amblyopia studies.

## Toward sex-aware research in amblyopia

It is time to shift the perspective on sex in amblyopia research to treating it as a biological variable that may influence neural plasticity mechanisms involved in the etiology and treatment of amblyopia. Modern neuroscience research recognizes that biological diversity is informative for understanding the neural basis of disorders and their treatments, and funding agencies now expect sex-aware analysis (NIH SABV, CIHR guidance). The field is primed to move from experimental designs that control for sex to ones that interrogate it’s impact on visual plasticity, amblyopia and its treatment (Figure 1). These changes are important for understanding the whole child because amblyopia is associated with a wide range of adverse systemic and mental health outcomes (Bountziouka et al., 2021; Lee et al., 2022, 2026; Chen et al., 2023; Wagner et al., 2024), including mental health risks that are more common in males (McKenzie et al., 2009; Bekmez et al., 2020).

To understand the basic mechanisms of visual cortical plasticity, studies need to routinely include both sexes, avoid pooling data, and use experimental designs with sufficient power to identify sex effects on plasticity. This will be especially important when examining molecular mechanisms that studies in other neural systems have already identified as differentially involved in regulating plasticity in females and males (e.g., Jain et al., 2018; Le et al., 2024). The influence of hormonal modulation and X-linked genes, especially those that escape inactivation in females, on critical-period plasticity remains an obvious gap that needs to be filled. Fundamental questions to address include the influence of sex on the onset and duration of critical-period plasticity in the visual cortex, as well as on the stabilization and durability of experience-dependent changes. These questions span levels of analysis from molecular mechanisms to physiology, anatomy and behavior.

Preclinical models of amblyopia treatments also need to use sex-aware experimental designs. For example, studies need to explicitly report the sex of all subjects and test for the influence of sex on monocular deprivation, environmental enrichment, and binocular recovery, as has been done with stress models of visual plasticity (Liu et al., 2020; Li et al., 2022). Animal models are uniquely suited to longitudinal studies that track recovery and relapse. Obvious questions about plasticity, durability, and recovery that need to be addressed include whether there are sex differences in ocular dominance recovery and in the reopening of critical periods. Furthermore, the role of hormones, especially estrogen, on visual cortex plasticity and recovery is an important yet understudied area (Sengupta et al., 2019).

For clinical and translational research, important steps include: pre-specifying sex as a biological variable to ensure the experimental design is appropriate for testing hypotheses; standardizing the collection of sex/gender information to enable comparison across studies; and sex-disaggregated reporting to enable comparison of outcomes. It will be important to design studies with sufficient power to stratify the data by age and sex, since the timing of plasticity windows is likely to differ between females and males. Amblyopia research can benefit from recommendations on how to increase our understanding of the influence of sex and gender, developed for neuroscience studies of mental health disorders and neuroimaging (DeCasien et al., 2022; Wierenga et al., 2024) that will contribute to the goal of treating the whole child (Birch and Kelly, 2023).

Importantly, in human studies, observed sex differences may reflect not only biological factors but also gender-related influences. Subtle differences in early visual experience could arise from social and environmental factors, such as differences in play, visual engagement, or parental expectations for girls and boys. Similarly, treatment adherence and visual outcomes may be shaped by caregiver behavior, including the consistency with which patching or other therapies are implemented. These influences may vary across cultural contexts and are often not measured in clinical studies. While such factors do not diminish the importance of biological mechanisms, they complicate the interpretation of sex effects in human amblyopia research and underscore the need for study designs that can better disentangle biological and environmental contributions.

Ultimately, these steps will help move amblyopia research toward a more precise understanding of how biological variability influences treatment response, supporting efforts to tailor interventions for diverse patient populations. For example, if sex-dependent differences in plasticity timing or stabilization are confirmed, treatment strategies such as the timing of patching initiation, the daily duration of occlusion therapy, or the intensity and duration of binocular therapy could potentially be adjusted to optimize recovery and reduce the risk of regression.

These priorities span the full research pipeline, from basic mechanisms of visual cortical plasticity to preclinical models and clinical studies (Figure 1). Amblyopia research is well-positioned to lead sex-aware plasticity research because the neural mechanisms are well understood, multiple effective treatments exist, and visual outcomes are measurable and standardized. As a result, amblyopia provides a uniquely tractable model for advancing sex-aware neuroscience, with the potential to reveal fundamental principles of developmental plasticity while improving treatment strategies for children with visual disorders.
